# RETRACTION: The Kinesin Light Chain‐2, a Target of mRNA Stabilizing Protein HuR, Inhibits p53 Protein Phosphorylation to Promote Radioresistance in NSCLC

**DOI:** 10.1111/1759-7714.70380

**Published:** 2026-08-24

**RETRACTION**: S. Qiao , Y. Jiang , N. Li and X. Zhu , “The Kinesin Light Chain‐2, a Target of mRNA Stabilizing Protein HuR, Inhibits p53 Protein Phosphorylation to Promote Radioresistance in NSCLC,” Thoracic Cancer 14, no. 16 (2023): 1440–1450, 10.1111/1759-7714.14886.37055376 PMC10234785

The above article, published online on 13 April 2023 in Wiley Online Library (wileyonlinelibrary.com), has been retracted by agreement between the journal Editor‐in‐Chief, Tateaki Naito; and John Wiley & Sons Australia, Ltd. The retraction has been agreed following concerns raised by a third party. An investigation identified several instances of panel duplication within Figures 1a and 1b, duplication of western blot bands within Figure 4l, and further duplication of western blot bands between Figures 3e and 3f. The authors did not respond to invitations to comment on the duplications or provide supporting data. The editors consider the results and conclusions to be compromised. The authors did not respond to the retraction notice.

